# Management of bipolar depression with lamotrigine: an antiepileptic mood stabilizer

**DOI:** 10.3389/fphar.2015.00242

**Published:** 2015-10-23

**Authors:** Kedar S. Prabhavalkar, Nimmy B. Poovanpallil, Lokesh K. Bhatt

**Affiliations:** Department of Pharmacology, Dr. Bhanuben Nanavati College of Pharmacy, Mumbai, India

**Keywords:** lamotrigine, focal epilepsies, bipolar disorder, mood destabilization, mania

## Abstract

The efficacy of lamotrigine in the treatment of focal epilepsies have already been reported in several case reports and open studies, which is thought to act by inhibiting glutamate release through voltage-sensitive sodium channels blockade and neuronal membrane stabilization. However, recent findings have also illustrated the importance of lamotrigine in alleviating the depressive symptoms of bipolar disorder, without causing mood destabilization or precipitating mania. Currently, no mood stabilizers are available having equal efficacy in the treatment of both mania and depression, two of which forms the extreme sides of the bipolar disorder. Lamotrigine, a well established anticonvulsant has received regulatory approval for the treatment and prevention of bipolar depression in more than 30 countries worldwide. Lamotrigine, acts through several molecular targets and overcomes the major limitation of other conventional antidepressants by stabilizing mood from “below baseline” thereby preventing switches to mania or episode acceleration, thus being effective for bipolar I disorder. Recent studies have also suggested that these observations could also be extended to patients with bipolar II disorder. Thus, lamotrigine may supposedly fulfill the unmet requirement for an effective depression mood stabilizer.

## Introduction

Management of bipolar disorders has given main focus on the treatment of acute mania, while neglecting the treatment of bipolar depression, both of which constitutes the extreme poles of the bipolar disorder (Podawiltz, 2012). However, greater interests in the therapeutic strategies that address the two extremities of the bipolar illness has resulted from the ever increased recognition of the importance of bipolar depression (Judd et al., 2002, 2003). Bipolar depression is considered to be a chronic illness and its episodes cause a great deal of suffering for patients as well as their family members (Bowden, 2010). Patients with bipolar depression are currently treated with medications such as mood stabilizers lithium, valproate and carbamazepine, or antidepressants (Yatham et al., 1997). Bipolar disorder which is also known as maniac-depression can be precisely described as a medical illness that is treatable and is marked by extreme changes in mood, thoughts, energy and behavior that can alternate between the poles of mania and depression, which could probably last for hours, days, weeks, or even months (Grande et al., 2015). Mood swings that accompany patients with bipolar disorder can be severe which could range from deep despair to extremes in energy having experience of “highs” and “lows” of the illness, which usually begins in the late adolescence appearing as depression during teen years. It could also be initiated in the early childhood or even as late as in the 40 and 50s (Ketter and Calabrese, 2002). Lamotrigine [3,5-diamino-6 (2,3-dichlorophenyl-1,2,4-triazine)] is an established anticonvulsant drug. It is approved for the treatment of focal epilepsies in the presence or absence of secondary generalization (Cheung et al., 1992).

Over the past few years, lamotrigine has also been found effective in the treatment of bipolar disorder being more efficacious in treating the depressive phase of the illness (Bowden et al., 1999; Reid et al., 2013). For the better understanding of the actions of currently available treatment options available, a new nomenclature for the bipolar disorder has been proposed in which “above baseline” comprises of events of mania, hypomania, and mixed state while depression and subsyndromal depression forms “below baseline.” On this basis, mood stabilizers could be categorized into two classes as Class A (mania mood stabilizers) and Class B (depression mood stabilizers; Ketter and Calabrese, 2002; Henry and Etain, 2010). For better understanding, depression mood stabilizers can be defined as the agents having the ability of stabilizing mood from “above baseline” with causing depression, while mania mood stabilizers could be defined as the agents capable of stabilizing mood from “below baseline” without switching back to mania (Herman, 2004). This novel nomenclature helps o serve the purpose of giving more focus to areas of unmet need in the management of bipolar disorder. There is an urgent need of the depression mood stabilizers for the treatment of bipolar depression as the conventional antidepressants that are used commonly for the acute treatment of bipolar depression fails the requirements of the definition of “depression mood stabilizers” mainly because of their tendency to cause mood destabilization by inducing switches to mania or episode acceleration (Post et al., 1997; Henry et al., 2001; Ketter and Calabrese, 2002; Sienaert et al., 2013). Lamotrigine possess the unique characteristic that differentiates it from the other mood stabilizers and anticonvulsants in its efficacy in bipolar disorder, as it exerts a positive effect on the corticolimbic network function, which is a resultant of abnormal activities of the circuits in bipolar depression (Reid et al., 2013). Lamotrigine is currently known for treating the depressive phase of bipolar I disorder (Bowden et al., 1999; Large et al., 2009). Although prevention of the relapse of depression in bipolar I disorder by lamotrigine monotherapy has been well demonstrated in the long-term studies, date regarding the same for the treatment of bipolar II disorder is scare (Bowden et al., 2003; Calabrese et al., 2003a). However, recent studies have suggested the long-term effectiveness of lamotrigine in treating patients with treatment-resistant bipolar II depression, with having obtained higher recovery rates from antidepressant augmentation with lamotrigine (Nierenberg et al., 2006; Sharma et al., 2008).

## Lamotrigine: A Depression Mood Stabilizer

Lamotrigine, an anticonvulsant drug is one such widely studied medication whose therapeutic efficacy has been extended even beyond its use as a mood stabilizer (Bowden and Singh, 2012). Lamotrigine monotherapy is recently used to control the depressive symptoms as observed in bipolar disorder suggesting its profound role in the management of bipolar depression (Ketter et al., 2008; Yatham et al., 2009). Lamotrigine is used primarily as an anticonvulsant for the treatment of generalized and partial seizures and is effective for treating focal epilepsies in the presence or absence of secondary generalization (Cheung et al., 1992; Kwan and Brodie, 2001; Bazil, 2002; Goldenberg, 2010). Antiepileptic activity of lamotrigine is mainly attributed to the inhibition of the voltage-sensitive sodium channels of the neuronal membrame, inhibition of the release of the excitatory amino acids such as glutamate and aspartate, and blockade of the calcium-channel (Cheung et al., 1992; Xie et al., 1995; Cunningham and Jones, 2000). The exact mechanism by which lamotrigine acts as a mood stabilizer remains unclear. Over several decades, there has been increased interest in the treatment of bipolar disorder by using medications such as carbamazepine and oxcarbazepine, which are the established antiepileptic agents as well as new generation antiepileptics such as lamotrigine and topiramate along with certain most widely used antidepressants were known to exhibit calcium-channel-blocking properties that are relevant to the pathophysiology of epilepsies. This property is also desired for alleviating depressive symptoms. Lamotrigine, however stands distinctive from the other antidepressants for treating bipolar depression in its cellular and molecular effects and in comparison with placebo, lamotrigine is considered as superior as it prolongs the time to intervention for depression (Grunze and Walden, 2002; Calabrese et al., 2003a,b). Even though mood stabilizing properties are also exhibited by other anticonvulsant drugs, several trials have shown lamotrigine to be outstandingly effective in the prevention or amelioration of bipolar depression in patients experiencing episodes of major depression as lamotrigine possess very low propensity for inducing switch to mania, which is major limitation with the use of other conventional antidepressants and also for preventing episodes of depression in patients diagnosed with rapid cycling bipolar disorder (Calabrese et al., 1999a,b, 2000, 2001).

## Bipolar Disorder in Bipolar I Disorder

Efficacy of lamotrigine was evaluated in subjects with bipolar I depression in a double-blind, parallel-group, multicenter trial (Calabrese et al., 1999a). In this study, lamotrigine 50 mg/d (*n* = 66), lamotrigine 200 mg/d (*n* = 63), or placebo (*n* = 66) were given to the patients showing 17-item HDRS score ≥18. After 7 weeks of treatment, primary measures were not significantly different secondary measures, had a significantly superior response rate on mean ± SD observed scores on 17-item HDRS, CGI-Severity of Illness, and CGI-Improvement in lamotrigine 200 mg/d group. In the four other studies subjects diagnosed with bipolar I disorder (Calabrese et al., 1999a; SCA40910 and SCA309241 Calabrese et al., 2008) were treated with lamotrigine 200 mg/d or placebo. 17-item HDRS total scores or the MADRS was not significantly different in treated or placebo, with the exception of 1 trial (Calabrese et al., 1999a). However, CGI-Improvement score was significantly greater with lamotrigine treated group in two studies (Calabrese et al., 1999a; SCA40910 and SCA309241 Calabrese et al., 2008). Amann et al. (2011) suggested that sufficient evidence are not available to recommend use of lamotrigine in the treatment of rapidly-cycling bipolar I.

## Lamotrigine in Bipolar II Disorder

Many prior studies have been focused on the efficacy of lamotrigine monotherapy in preventing the depressive relapse in bipolar I disorder and rapid-cycling disorder. Bipolar II disorder is characterized by one or more episodes of major depression which is often accompanied by atleast one hypomania episode. In contrast to this, bipolar II depression is associated with either one or more maniac or depressive episodes (mixed episodes) which may alternate from depressive episode lasting for several weeks or months to intense maniac symptoms lasting for about the same duration. In patients with bipolar II disorder, the clinical data for the long term use of lamotrigine is very limited (Bowden et al., 2003). Patients suffering from the depressive phase of the bipolar disorder were suggested with adjunctive lamotrigine treatment, the usage of which was considered to be safe and well tolerated. Lamotrigine therapy showed significant response in comparison to placebo in severely ill patients in a recent meta-analysis conducted (Geddes et al., 2009). It was found that antidepressant augmentation with lamotrigine was reported to show higher rates of recovery in treatment-resistant bipolar I or II disorder. Results that were obtained from related studies conducted earlier gave sufficient evidence for the long-term effectiveness of lamotrigine adjunctive therapy in the management of treatment-resistant bipolar II depression (Sharma et al., 2008). In comparison with bipolar I disorder, higher suicidal tendency and psychosocial impairment was observed in bipolar II disorder. The longitudinal course of bipolar II disorder is dominated by recurrent depressive episodes and residual symptoms of depression, which has a potential to exert strong influence on the psychosocial functioning levels. Owing to this, the clinical output of bipolar II disorder can be improved by the proper long-term management of the depressive symptoms (Endicott et al., 2008; Vieta and Suppes, 2008). With this respect, a naturalistic 52-week follow-up study was conducted for evaluating the long-term effectiveness of lamotrigine adjunctive therapy for bipolar II depression, in the absence of treatment-related mania or hypomania. The results of which showed that the severity of depression was significantly reduced throughout 52-weeks in bipolar II disorder. During the initial 12-week treatment, the rate of response was found to be 64.5% of the adjunctive lamotrigine, when tested in the same population. Depression was improved during the initial treatment phase and maintained for about 1 year (Chang et al., 2010). Management of acute phase of bipolar depression was found to be ineffective by lamotrigine monotherapy as per the results obtained from the five randomized trials whereas, addition of lamotrigine to lithium in treating patients with acute bipolar depression was found to effective on the basis of the results of a recently conducted randomized trial (Calabrese et al., 2008; van der Loos et al., 2009). Augmentation of atypical antipsychotics or mood stabilizers with lamotrigine is expected to produce additive or synergistic effects through modulation of sodium channel or serotonergic regulation (Bourin et al., 2009). The results obtained from an animal study using sodium channel opener, lamotrigine showed antidepressant effects due to two different mechanisms such as inactivation of sodium channels and monoaminergic neurotransmission suggesting different mode of antidepressant activity (Prica et al., 2008). Adjunctive use of lamotrigine may be more beneficial in terms of safety and tolerance as compared to lamotrigine monotherapy, since patients already receiving mood stabilizers could easily avail a slower titration of lamotrigine (Joe et al., 2009).

## Lamotrigine in Bipolar Depression Augmentation

In various clinical trials lamotrigine use as an augmentation agent was explored for bipolar disorder with depressive episodes not responsive to typical mood stabilizers. In a randomized, double-blind, 7-week pilot trial, lamotrigine augmentation was compared with citalopram augmentation for bipolar I and II depression (Schaffer et al., 2006). Total MADRS score difference was significant for both lamotrigine and citalopram. Nierenberg et al. (2006) compared lamotrigine, risperidone, and inositol in a 16-week, randomized, equipoise-stratified study in subjects with bipolar I or II disorder. Although no significant difference was found between groups for primary outcome, subjects in lamotrigine group remained in the randomized phase significantly longer compared to other groups. van der Loos et al. (2009) in an 8-week, multicenter, double-blind, randomized, placebo-controlled trial studied lamotrigine augmentation to lithium in non-lithium responding subjects, for treating bipolar depression. This study evaluated efficacy of lithium plus lamotrigine for the treatment of acute bipolar depression. MADRS score change was significantly different between groups. Further, subjects were more responsive in lithium plus lamotrigine treated group compared to placebo. In a recent study, Kagawa et al. (2014) reported that in treatment-resistant depressive plasma lamotrigine concentration of 12.7 μmol/L may be a threshold for better therapeutic response.

## Effect of Lamotrigine on 5-HT Receptor

The antidepressant drug lamotrigine is found to be therapeutically more effective in the treatment of depressive phase of the bipolar disorder than in treating hypomania or mania (Bourin et al., 2005). The use of lamotrigine as an augmentation drug was suggested from the evidence of the efficacy of the drug in the treatment of major depressive disorders, which also includes refractory unipolar depression (Frye et al., 2000; Barbee and Jamhour, 2002; Rocha and Hara, 2003). The onset of antidepressant action accelerated when administered in combination with other classical antidepressants (Normann et al., 2002). Pilot studies have reported the augmentation therapy of lamotrigine with SSRI’s (fluoxetine and paroxetine) to be well tolerated and far more superior in efficacy than that of SSRI monotherapy (Normann et al., 2002; Barbosa et al., 2003).

In a study, various *in vivo* evaluations in various regions of the brain were conducted to understand the effect of lamotrigine on serotonin (5-hydroxytryptamine, 5-HT)_1A_-receptor-mediated adenyl cyclase responses in various brain areas in the mice. The results, which were obtained thereafter, suggested a new pathway which reflected the therapeutic efficacy of various classes of mood stabilizers. This *in vivo* study was therefore successful in explaining lamotrigine’s mode of action that was eventually a result of downregulation of cortical 5-HT_1A_-receptor-mediated adenyl cyclase responses. Thus showing significant role of 5-HT in the physiopathology of maniac depressive disorder (Vinod and Subhash, 2002). For quantifying 5-HT_1A_, 5-HT_1B_, and 5-HT_2A_ m-RNA levels in the hippocampus and dorsal prefrontal cortex, the technique of in situ hybridization was used, as a part of a recent study of subjects with a history of bipolar disorder. The result of the same indicated that these subjects showed highly altered 5-HT receptor m-RNA expression giving more evidence to support the involvement of 5-HT receptors in the mechanism of action of lamotrigine in bipolar disorders (Lopez-Figueroa et al., 2004).

Shim et al. (2013) performed electrophysiological studies in the rat brain to evaluate modulation of the antidepressant-like effects of sustained administration of carisbamate and lamotrigine on monoaminergic mystems. They concluded that 5-HT firing in the DRN is decreased but 5-HT transmission in the forebrain is enhanced by sustained carisbamate and lamotrigine administration.

One of the biggest challenges faced in the studies and evaluation of mood stabilizers was the development of a valid and satisfactory animal model for bipolar disorder as it comprised of complex interplay of episodes of mania, depression, euthymia and mixed states. The animal models that have been proposed was found only to be partially matching the bipolar illness and in such case modeling of either depression or maniac behavior could be achieved, but not both (Machado-Vieira et al., 2004). The FST, as compared to all the other models was found to be a simpler model (Porsolt et al., 1977) and match most of the required criteria of the illness and it has been used extensively in the investigations of mechanisms of antidepressant actions (Redrobe and Bourin, 1999a). FST is also one such sensitive model to the compounds acting on the 5-HT system (Redrobe et al., 1996; Redrobe and Bourin, 1997), which was of great help in the association studies that involved lamotrigine along with the most specific brain-penetrating ligands for 5-HT_1A_ and 5-HT_1B_ receptors currently available to study the possible interaction between the two and also to investigate the potential antidepressant-like action of lamotrigine (Redrobe and Bourin, 1999b, Tatarczynska et al., 2004). 5-HT1 receptor subtype are known to exist as “autoreceptors” at the presynaptic level controlling the release of 5-HT and also exist as “heteroreceptors” at the postsynaptic level, coupled to adenylate cyclase in controlling the release of other neurotransmitters (Sarhan and Fillion, 1998). The subsequent studies showed that only a high affinity postsynaptic 5-HT_1A_ receptor agonists, a presynaptic and postsynaptic 5-HT_1A/1B_ receptor antagonists and to a lesser extend postsynaptic 5-HT_1A/1B_ agonists were able to enhance the antidepressant-like effect of lamotrigine in FST. All the data obtained from the study showed significant decrease in the immobility time of lamotrigine when used in combination with the various 5-HT ligands indicating the involvement of postsynaptic 5-HT receptors in activity of lamotrigine. The antidepressant-like effect of lamotrigine was also known to be produced by the neurotransmitters regulated by postsynaptic 5-HT receptors (Bourin et al., 2005). Another study on the effect of 5HT transmission on lamotrigine reported carbamazepine and lamotrigine to be the only anticonvulsant drugs to result in elevated extracellular 5-HT concentrations by inhibiting both *in vitro* and *in vivo* 5-HT uptake (Clifford et al., 1998). Cortical 5-HT_1A_ receptors mediated downregulation of responses also suggests another possible mechanism of action of lamotrigine (Vinod and Subhash, 2002).

## Effect of Lamotrigine on the Noradrenergic Receptors

Evaluation of the antidepressant-like effect of lamotrigine in the mouse forced swimming test along with the involvement of noradrenergic system was done in a recent study, the results of which showed convincing evidence in the reduction of immobility time in the forced swimming test, the effect of which is mediated by an interaction with the noradrenergic system, probably with postsynaptic α_1_- and α_2_-adrenoreceptors (Petit-Demouliere et al., 2005; Consoni et al., 2006). For studying the involvement of noradrenergic system in antidepressant-like effect of lamotrigine, a selective inhibitor of the enzyme tyrosine hydroxylase, α-methyl-*p*-tyrosine was used which is a rate-limiting enzyme in the noradrenaline and dopamine synthesis. It was found that when higher dose this inhibitor was given in combination with lamotrigine, it produced sedative effect, which was considered to be responsible for the reversal of the antidepressant-like effect of lamotrigine. Such reversal of the anti-immobility effect was also found at lower doses of α-methyl-*p*-tyrosine (Kaster et al., 2007). It was also demonstrated that in mice, α-methyl-*p*-tyrosine without affecting the levels of serotonin, reduced levels of dopamine and noradrenaline (57 and 53% respectively; Mayorga et al., 2001). Moreover, a temporary reversal in the antidepressant response to desipramine, mazindol, and mirtazapine was produced by the acute administration of α-methyl-*p*-tyrosine (Delgado et al., 1993; Miller et al., 1996). Lamotrigine was also reported to inhibit the synaptosomal uptake of serotonin, noradrenaline, and dopamine in the rat brain which could be an indication that lamotrigine indirectly affects the noradrenergic system, by the release of noradrenaline, which could further result in interaction with the receptors (Southam et al., 1998).

The behavioral models of depression depicting antidepressant-like responses of certain drugs are seemed to be influenced by α_1_- and α_2_-adrenoreceptors (Kitada et al., 1983; Danysz et al., 1986; Masuda et al., 2001). When mice were pretreated with prazosin and yohimbine, it reversed the decrease in the immobility time evoked by lamotrigine suggesting the underlying actions of these receptor subtypes in the forced swimming test. In another study, yohimbine could reverse the antidepressant-like effect produced by clonidine in the forced swimming test of mice whereas, there was prevention in the antidepressant-like action of desipramine when mice were pretreated with prazosin (O’Neill et al., 2001). Furthermore, when mice were pretreated with either phenylephrine or clonidine and given in combination with subeffective doses of lamotrigine, in the mice forced swimming test, it produced synergistic antidepressant-like effects suggesting the involvement of α_1_- and α_2_-adrenoreceptors activation in the mechanism of action of lamotrigine (Kitada et al., 1983). The immobility time in the forced swimming test in rats and mice was also found to be decreased by clonidine, an α_2_-adrenoreceptor agonist (Cervo and Samanin, 1991; O’Neill et al., 2001). A strong reduction in the locomotor activity which was produced by the administration of adrenergic drugs phenylephrine and clonidine was already reported in the earlier study. However, the more recent study conducted on the involvement of noradrenergic system in the antidepressant-like action of lamotrigine reported that inspite of these adrenergic drugs showing sedative effects (Sukul et al., 1988; Cervo and Samanin, 1991; Hascoet et al., 1991), they potentiated the antidepressant-like effect of lamotrigine. The results of this study also suggested the activation of the postsynaptic α_1_- and α_2_-adrenoreceptors is due to the enhancement of noradrenaline levels by lamotrigine (Kaster et al., 2007).

## Efficacy of Lamotrigine: Dysfunction of Neural Network in Bipolar Depression

A better understanding of the mechanisms of action of mood-stabilizing agents forms a basis of the mechanisms underlying the pathology of bipolar disorder. For the better understanding of the efficacy of lamotrigine, assessment of neural activity in different areas of the brain was undertaken in healthy human volunteers to study the effects of the drug by activating specific neural circuits using transcranial magnetic stimulation (TMS) which was used in combination with functional magnetic resonance imaging (fMRI) for monitoring the response. This is a significant non-invasive method for the assessment of motor cortex excitability which also helps in the stimulation of the cerebral cortex. This method is also found to be useful in the pharmacological examination of neuroactive drugs on specific brain circuits (Li et al., 2004; Large et al., 2009). It was observed from the previous studies which used TMS (Manganotti et al., 1999; Tergau et al., 2003), it was expected that lamotrigine 325 mg activated the abductor digiti minimi muscle by increasing the stimulus following motor cortex stimulation. TMS-induced motor cortical circuits activation was observed beneath the stimulator coil in the presence of the drug. Lamotrigine was also found to show facilitatory effect on the blood-oxygen-level-dependant (BOLD) response to TMS of the prefrontal cortex. Application of TMS to the motor cortex showed that with the reduction in the excitability of the motor cortex, reduction of the BOLD response was consistent, which was in line with the sodium channel blocker effects (Paulus et al., 2008). TMS was applied to the prefrontal cortex in the second part of the study that elicited BOLD response in the corticolimbic brain areas such as hippocampus and orbital frontal gyrus thereby showing increased excitability of prefrontal cortex.

The detailed investigations in the study showed that anticonvulsant activity of the antiepileptic drugs such as lamotrigine and valproic acid is conferred to the inhibition of the brain activation caused by the reduced excitability of the motor cortex induced by TMS stimulation. However, lamotrigine exerts a different action in the prefrontal corticolimbic system on TMS stimulation which shows that lamotrigine differs from valproic acid in molecular mechanisms of action as increased TMS-induced BOLD response was observed in corticolimbic circuits when TMS was applied over the dorsolateral prefrontal cortex (Li et al., 2006). This contrasting neurological effects of lamotrigine on the excitability of prefrontal and motor circuits shows some relevance to the drug’s efficacy in the treatment of bipolar disorder and epilepsy respectively (Li et al., 2004). Thus, lamotrigine, anticonvulsant drug, was found to exert a positive effect on the activity of the corticolimbic circuits in patients with bipolar disorder.

Further studies for highlighting the facilitatory effects of lamotrigine on the activity of the corticolimbic system was done to investigate the *in vitro* activity of the hippocampus in response to the drug (Large et al., 2009). It was very much necessary to study the effects of the drug on the brain neural network, rather than on the individual neurons (Xie et al., 1995). For this purpose, the CA3 region of the hippocampus was chosen for studying the effects of lamotrigine on oscillatory field potentials, manifested at low concentrations of kainic acid resulted in network oscillations (Fisahn, 2005), which was believed to be able of mimicking the physiological neural network activity, responsible for the encoding and retrieval of memory in the hippocampus (Mann and Paulsen, 2005). In the hippocampus, it was observed that the power of the gamma oscillations induced by kainic acid was enhanced significantly by lamotrigine at concentrations consistent with the therapeutic range (3–10 μM), in contrast to valproic acid which had no effect at its therapeutic concentration of 100 μM. Decreased neural activity was seen with higher concentrations of lamotrigine (100 μM). The results obtained from the neuronal network studies provided sufficient evidence of the facilitatory effects of lamotrigine in healthy volunteers and in patients with bipolar disorder (Large et al., 2009). Thus, along with the activity of the corticolimbic circuits lamotrigine also exerts a positive effect on the neural network activity.

Based on this, a hypothesis was drawn that neuroplasticity dysfunction and cellular resilience formed an important component of the bipolar disorder. With use of clinically effective mood stabilizers such as lithium and valproic acid this approach was utilized (Bachmann et al., 2005; Schloesser et al., 2008). Efficacy of this approach has suggested a various intracellular signaling elements like glycogen synthase kinase 3 ß(GSK3 ß), extracellular-signal-regulated kinase (ERK)/mitogen-activated protein kinase (MAPK) or protein kinase C as the main targets. However, it was found that lamotrigine differs markedly from valproic acid and lithium in treating bipolar disorder as none of the targets for bipolar disorder seemed to be modulated by lamotrigine, showing greater efficacy for the prevention/amelioration of episodes of depression rather than mania (Large et al., 2009).

There are several cellular and molecular actions of lamotrigine that may contribute to its action in bipolar disorder (Xie and Hagan, 1998). The main mechanism by which lamotrigine emerged as a well-established anticonvulsant is due to the inhibition of neuronal hyperexcitability and modification of synaptic plasticity via voltage-dependant inhibition of neuronal voltage-activated Sodium channels and possible high voltage-activated Calcium channels, as a result of which excessive neurotransmitter release in the brain is reduced (Xie et al., 1995). However, the reason for this mechanism of lamotrigine to exert its efficacy against depressive phase of the bipolar disorder is quite unclear.

## Lamotrigine in Bipolar Disorder: Interactions with the Molecular Targets

As discussed earlier, some important molecular targets such as serine/threonine kinase, GSK3 which forms a central component of the Wnt signaling pathway as well as in the other pathways such as phosphoinositide 3-kinase (PI3K)/Akt intracellular signaling pathways are known for its important role played in the regulation of multiple cellular processes such as apoptosis, metabolism, proliferation, differentiation, synaptogenesis, and resilience/cellular plasticity (Large et al., 2009). In 1996, GSK3 pathway was first linked to bipolar disorder from the finding that lithium is a direct inhibitor of GSK3 and it directly as well as indirectly inhibited GSK3 by increasing the phosphorylation of Akt, which inturn is responsible for the phosphorylation of Ser^9^ (Phiel and Klein, 2001; De Sarno et al., 2002). However, lamotrigine indirectly acts by attenuation of staurosporine and heat-shock-induced caspase 3 activity in a cell line responsible for over expression of GSK3 (Bijur et al., 2000). One interesting fact from a study was that when cerebral cortical cells or neuroblastoma was exposed to lamotrigine, glycogen synthase kinase-3β was inhibited which inturn increased the activity of an oxidizing enzyme, glutathione *S*-transferase (Hayes and Strange, 2000; Strange et al., 2001; Bakare et al., 2009). Lamotrigine is reported to show no significant effects on PKC activity, which in contrast to valproic acid and lithium are reported to indirectly result in reduction of the levels and activity of PKC isoforms α and ε in the prefrontal cortex and hippocampus of the rat, which is not a direct effect on PKC (Lenox et al., 1992; Watson and Lenox, 1996). Comparative analysis of the effects of four main mood stabilizers including valproic acid, lithium, carbamazepine and lamotrigine has proved that there was no activation of the ERK/MAPK signaling pathway by lamotrigine (Di Daniel et al., 2005) whereas lithium and valproic acid was found to cause activation of ERK/MAPK pathway in the cell lines and rat primary cortical neurons also in the hippocampus and prefrontal cortex of the rat (Yuan et al., 2001; Einat et al., 2003).

Bipolar disorder is characterized by hyperglutamatergic neurotransmission and by upregulated arachidonic acid cascade, which forms a new profound therapeutic target in the treatment of the disorder (Michael et al., 2003; Zarate et al., 2003; Clinton and Meador-Woodruff, 2004; Cherlyn et al., 2010). The mechanism of NMDA receptor-mediated arachidonic acid signaling which is responsible for triggering a bipolar disorder is through glutamatergic neurotransmission, which involves *N*-methyl-D-aspartate receptors (NMDAR’s). These receptors allow the extracellular flow of calcium into the cell, which causes the membrane phospholipid to selectively release arachidonic acid, via the activation of cPLA_2_ type IV (calcium-dependant cytosolic phospholipase A_2_-IV; Epolia et al., 2012). Studies show that in bipolar disorder, associated with the above mechanisms, lamotrigine was found to cause interference with the glutamatergic neurotransmission that involved the NMDA receptors. Lamotrigine reduces the presynaptic neuronal depolarization by acting on the voltage-gated sodium and calcium channels thereby reducing the release at the excitatory synapse (Xie and Hagan, 1998; Cunningham and Jones, 2000; Sitges et al., 2007a,b) as well as through blockade of serotonergic receptors (Ketter et al., 2003). However, lamotrigine does not produce any effect on the resting membrane potential, excitatory neuronal transmission of low-frequency or neuronal excitability (Xie and Hagan, 1998), even the release of glutamate in the hippocampal region of the freely moving rats (Ahmad et al., 2004), under basal conditions. Thus lamotrigine, like many other approved mood-stabilizers causes inhibition of the arachidonic acid signaling mediated by the NMDA receptors thereby bringing about down-regulation of brain metabolic arachidonic acid cascade in the rat brain, initiated by NMDA (Rapoport and Bosetti, 2002; Rapoport et al., 2009). Moreover, the post mortem BD rat brain showed consistent glutamatergic state with up-regulated arachidonic acid cascade and NMDA receptor signaling markers which includes COX-2 (cyclooxygenase), PLA_2_, and PG E synthase (prostaglandin E synthase) responsible for the conversion of arachidonic acid to pro-inflammatory Prostaglandin E_2_, greater expression of vesicular glutamate transporter I, as well as elevated levels of protein and m RNA in the rat frontal cortex (Hashimoto et al., 2007; Rao et al., 2007, 2010; Kim et al., 2011). It was also found that with chronic lamotrigine treatment when administered at therapeutically relevant doses, COX-2 protein and m-RNA was found to be decreased in the rat brain, reduced DNA binding activity of NF-κB and decreased PG-E_2_ and thromboxane (TXB_2_) concentrations (Lee et al., 2008). Lamotrigine was also found to suppress ketamine induced perceptual abnormalities in healthy human volunteers (Anand et al., 2000). Besides its efficacy in treating bipolar disorder by inhibition of the metabolic arachidonic cascade, lamotrigine has also been reported for its neuroprotective effects with respect to bipolar disorder arising out from its potential to increase the levels of mRNA and proteins of brain-derived neurotropic factor (BDNF) as well as by increasing the levels of anti-apoptotic factor B-cell lymphoma 2 (Bcl-2; Chang et al., 2009; Li et al., 2010). Lamotrigine was also used successfully in the experimental models of cerebral ischemia as well as in excitotoxicity induced by glutamate (Bacher and Zornow, 1997; Maj et al., 1998). In the rat brain, down regulation of BDNF induced by stress was also reversed by LTG (Frye et al., 2007).

## Limitations of Lamotrigine

After vigabatrin and felbamate, the third ever new generation antiepileptic to be commercialized was lamotrigine. However, out of these two drugs only lamotrigine for studied for its utility as a mood stabilizer, while the other two were not studied as after its commercialization as antiepileptics they showed idiosyncratic side-effects namely aplastic anemia and visual field problems which has limited their use for treating very special epilepsy cases. Undoubtedly, lamotrigine has given the best results as compared to all other third-generation anticonvulsants in the treatment of bipolar disorder being more therapeutically effective in treating the depressive episodes encountered in a bipolar disorder (Vieta, 2004). Despite of its proven importance in bipolar disorder, several limitations are also exhibited by lamotrigine as reported by some studies. The data obtained from a controlled clinical trial using placebo showed positive results for lamotrigine only in patients with bipolar II disorder. A double-blind placebo-controlled clinical trial was conducted confirmed that the doses ranging from 50 to 200 mg/day was found to be very effective in bipolar depression, however the efficacy as shown by the Hamilton depression scale was not significant. Lamotrigine was also reported to show negative results in acute mania as per the results obtained from two double-blinded placebo-controlled studies (Vieta, 2004).

Treatment of bipolar II depression with adjunctive lamotrigine may also be associated with a history of suicide-attempts and a number of depression-related prior hospitalization in case of poor response to the adjunctive lamotrigine therapy, as per the data from the 52-week follow-up study. In case of a history of previous hospitalization, an increased risk of re hospitalization, treatment resistance, and depressive relapse was reported (Souery et al., 2007; Lin et al., 2008). The risk of bipolar disorder may be well influenced by harm avoidance linked to genetic variations, which can also alter responses to the pharmacological treatment (Mandelli et al., 2009). The 52-week naturalistic study also reported cases of lamotrigine discontinuation among 8.3% of the study subjects owing to the treatment related adverse events (Chang et al., 2010). Inspite of its good tolerability profiles with lower incidences of weight gain, there is a fear of risk of Syndrome of Steven-Johnson, though very infrequent. Doses of lamotrigine ranging from 150 to 225 mg/day (Fatemi et al., 1997), which could also be raised up to 500 mg/day (Dichter and Brodie, 1996) can produce benign rash, reported to appear in 10% of the cases. However, at the time of initiation of the treatment, slow titration of the lamotrigine dose could decrease the inherent risk and incidence of rashes. In patients with bipolar disorder, lamotrigine however does not show any detrimental impact on the neurocognitive functions, thereby ensuring the safe and long-term use of lamotrigine in bipolar disorder (Daban et al., 2006). Further, in a recent review Parker and McCraw (2015) explored disconnect between the clinical efficacy and quantified efficacy in controlled trials. They conclude that iterative process should be encouraged between efficacy studies and clinical observation.

## Model for Management of Bipolar Depression using Lamotrigine

Various studies regularly reported partial rather than full response for lamotrigine in bipolar depression, both acute and maintenance. For depressive symptoms lamotrigine can serve as first choice drug for augmentation to a primary mood stabilizer for depressive symptoms. Since lamotrigine has certain limitations it can be supported with a second drug or non-drug intervention (Bowden and Singh, 2012). Further a recent study showed that lower lamotrigine serum concentrations resulted in therapeutic benefit in-patient with bipolar disorder (Unholzer and Haen, 2015).

## Conclusion

This review highlights the importance of understanding the different mechanisms and pathways contributing to the therapeutic use of the well-established antiepileptic drug lamotrigine in the management of bipolar disorder, being more effective in treating the depressive phase of the disorder as well as how it differs from other clinically relevant mood stabilizers, conventional antidepressants and anticonvulsants in exerting its efficacy over bipolar depression. A large double-blind study showed that lamotrigine monotherapy was far more superior over placebo in the patients presented with mild to very severe bipolar depression. It was also demonstrated that lamotrigine was used either as sole therapy or in adjunct with lithium or valproic acid in the treatment of bipolar depression, which is usually well tolerated. Data provided in this review also focus on the effectiveness of lamotrigine in alleviating bipolar depression without resulting in mood destabilization and in showing significant improvements in both depressive as well as maniac phases of the bipolar disorder. Prefrontal cortex of the brain which plays an important role in processing emotions as well as in reward processing has been shown to be disrupted in bipolar disorder, which was found to be overcome by lamotrigine by increasing BOLD-response in the hippocampus contrasting the effects of lamotrigine produced on the motor circuits which could provide future highlights in the improved therapeutic targets for bipolar disorder. The value of investigating the effects of psychoactive drugs on the neural circuits was also reviewed thereby providing better understanding of lamotrigine’s efficacy in treating bipolar depression. Lamotrigine is also shown to possess some limitation, encountered during the course of the treatment though infrequent. Thus, in comparison with the mood stabilizers which currently lack the ability to cause stabilization from “below baseline” lamotrigine is considered to be given a welcome addition to the treatment options available in the management of bipolar disorder and the emerging results from the previous studies also extends its usefulness in patients with bipolar II disorder and in rapid-cycling disorder, however more placebo-controlled trials would be required for the complete confirmation of the efficacy of lamotrigine, an antiepileptic mood stabilizer in the treatment of bipolar depression.

### Conflict of Interest Statement

The authors declare that the research was conducted in the absence of any commercial or financial relationships that could be construed as a potential conflict of interest.
